# Lipocalin 2 Enhances Migration and Resistance against Cisplatin in Endometrial Carcinoma Cells

**DOI:** 10.1371/journal.pone.0155220

**Published:** 2016-05-11

**Authors:** Tsutomu Miyamoto, Hiroyasu Kashima, Yasushi Yamada, Hisanori Kobara, Ryoichi Asaka, Hirofumi Ando, Shotaro Higuchi, Koichi Ida, David Hamisi Mvunta, Tanri Shiozawa

**Affiliations:** Department of Obstetrics and Gynecology, Shinshu University School of Medicine, 3-1-1 Asahi, Matsumoto, 390–8621, Japan; University of South Alabama Mitchell Cancer Institute, UNITED STATES

## Abstract

**Purpose:**

Lipocalin 2 (LCN2) is a secretory protein that is involved in various physiological processes including iron transport. We previously identified LCN2 as an up-regulated gene in endometrial carcinoma, and found that the overexpression of LCN2 and its receptor, SLC22A17, was associated with a poor prognosis. However, the functions and mechanism of action of LCN2 currently remain unclear.

**Methods:**

The LCN2-overexpressing endometrial carcinoma cell lines, HHUA and RL95-2, and LCN2-low-expressing one, HEC1B, were used. The effects of LCN2 on cell migration, cell viability, and apoptosis under various stresses, including ultraviolet (UV) irradiation and cisplatin treatment, were examined using the scratch wound healing assay, WST-1 assay, and Apostrand assay, respectively.

**Results:**

LCN2-silencing using shRNA method significantly reduced the migration ability of cells (p<0.05). Cytotoxic stresses significantly decreased the viability of LCN2-silenced cells more than that of control cells. In contrast, LCN2 overexpression was significantly increased cisplatin resistance. These effects were canceled by the addition of the iron chelator, deferoxamine. After UV irradiation, the expression of phosphorylated Akt (pAkt) was decreased in LCN2-silenced cells, and the PI3K inhibitor canceled the difference induced in UV sensitivity by LCN2. The cisplatin-induced expression of pAkt was not affected by LCN2; however, the expression of p53 and p21 was increased by LCN2-silencing.

**Conclusions:**

These results indicated that LCN2 was involved in the migration and survival of endometrial carcinoma cells under various stresses in an iron-dependent manner. The survival function of LCN2 may be exerted through the PI3K pathway and suppression of the p53-p21 pathway. These functions of LCN2 may increase the malignant potential of endometrial carcinoma cells.

## Introduction

Endometrial carcinoma is the fifth most common carcinoma in women worldwide [1]. The incidence and mortality rate of endometrial carcinoma is increasing in the United States (the SEER database) [2] and Japan [3]. Surgery is the first choice of treatment for early stage disease, and the outcome is generally preferable. Advanced disease is treated with surgery and chemotherapy such as AP (doxorubicin and cisplatin) and TC (paclitaxel and carboplatin) or with radiation [4]; however, the prognosis is limited. Although several molecular targeting therapies such as mTOR inhibitors have been attempted in the treatment of advanced or recurrent cases, their effects have not been satisfactory [5,6]. Therefore, a deeper understanding of the molecular mechanisms underlying the pathogenesis and progression of this cancer is needed for better management.

We previously searched differentially expressed genes in normal and neoplastic endometrial tissues using laser-captured microdissection and microarray analyses in order to identify new genes involved in endometrial carcinogenesis [7]. Consequently, we identified lipocalin 2 (LCN2) as a gene that was expressed at higher levels in endometrioid adenocarcinomas of the endometrium (EEC) than in normal endometria, as well as a step-wise increasing gene along with the progression of the disease from normal endometria, through endometrial hyperplasia, and to carcinoma. LCN2 is a 25kDa soluble and secretory protein that is also referred to as neutrophil gelatinase-associated lipocalin (NGAL) or 24p3. 24p3 was originally cloned from mouse kidney cells infected with SV40 [8]. Human NGAL, a homologue of mouse 24p3, was identified as a protein that formed a complex with a 92kDa gelatinase in neutrophils [9]. LCN2 is also known to act as an iron transporter [10]; it binds to cell surface receptors including solute carrier family 22 member 17 (SLC22A17) or megalin, and is transported into the cell [11, 12]. In acute bacterial infection, LCN2 mediates an innate immune response and inhibits bacterial growth by depriving of the iron-siderophore complex from bacteria [13]. Previous studies elucidated additional functions including the protective effects against degradation of MMP-9 [14], and the facilitatory effects of epithelial-mesenchymal transition [15]. We also reported that increases in the expression of LCN2 correlated with the enhanced invasion of extravillous trophoblasts [16].

We previously showed that the immunohistochemical expression of the LCN2 protein was increased in higher grade and advanced stage EEC [7], and the overexpression of LCN2 and SLC22A17 was an independent prognostic factor [17]. Furthermore, the forced expression of LCN2 enhanced the proliferation and invasion of endometrial carcinoma HEC1B and Ishikawa cells [7]. The up-regulation of LCN2 expression has also been reported in several other carcinomas, such as those in the esophagus, mammary glands and ovary [18–20], and LCN2 has been associated with the malignant potential of carcinoma cells. However, the functions of LCN2 in endometrial carcinoma cells, as well as the intracellular mechanisms involved remain undetermined.

In order to clarify the function of LCN2 in endometrial carcinoma cells, we herein focused on the effects of LCN2 on cell migration and cell survival under various stresses such as cisplatin and ultraviolet irradiation, which are typical DNA-damaging factors. We found that cell migration and resistance to serum starvation, cisplatin, and ultraviolet irradiation were enhanced by LCN2, suggesting that it confers malignant potential to endometrial carcinoma cells.

## Materials and Methods

### Culture of Endometrial Carcinoma Cells and Gene Transfer

The endometrial carcinoma cell line, HHUA [21] was purchased from the Riken Cell Bank (Cell No.RCB0658, Saitama, Japan) with the permission of Dr. Ishiwata at the Ishiwata Laboratory (Mito, Japan). Ishikawa [22] was kind gifts from Dr. H. Nishida (Kasumigaura Medical Center, Tsuchiura, Japan). The other endometrial carcinoma cell lines, HEC1A, HEC1B [23], KLE [24] and RL95-2 [25] were purchased from the American Type Culture Collection (Cat. No.HTB-112, HTB-113, CRL-1622 and CRL-1671, Rockville, MD).

HHUA cells, derived from well differentiated endometrial adenocarcinoma strongly expressing LCN2, were maintained in F-12 medium (Life Technologies, New York, NY) containing 15% fetal bovine serum (FBS, Equitech Bio., Texus, TX, USA). RL95-2 cells, derived from moderately differentiated endometrial adenosquamous carcinoma strongly expressing LCN2, were maintained in DMEM/F-12 medium (Life Technologies) containing 10% fetal bovine serum and 0.005 mg/ml insulin. In order to establish LCN2-silenced HHUA and RL95-2 cells, a pGFP-V-RS vector producing LCN2-specific short hairpin RNA (shRNA) (OriGene, Rockville, MD) was transfected into HHUA and RL95-2 by lipofection according to the supplier’s instructions (Lipofectamine 2000, Invitrogen, Carlsbad, CA). There were 4 types of shRNA for LCN2 (shRNA-1: 5'-tacaatgtcacctccgtcctgtttaggaa-3', shRNA-2: 5'-gagaaccaaggagctgacttcggaactaa-3', shRNA-3: 5'-agaacttcatccgcttctccaaatctctg-3', shRNA-4: 5'-tacctcgtccgagtggtgagcaccaacta-3'). Stably LCN2-silenced clones were then selected by puromycin (Sigma-Aldrich, St. Louis, MO). The same vector producing scramble (5'-gcactaccagagctaactcagatagtact-3') was transfected by the same method to establish control HHUA and RL95-2 cells. LCN2-overexpressing HEC1B cells and control HEC1B cells were established by the transfection of pCEP4-LCN2 or empty vector kindly provided by Dr. Kornelia Polyak [26], as described previously [7]. These cells were used in the following assays.

### RT-PCR

Endometrial carcinoma cell lines (Ishikawa, HHUA, HEC1A, HEC1B, KLE, and RL95-2) were subjected to RT-PCR. Total RNA extracted by TRIzol reagent (Invitrogen) according to the manufacturer’s instructions, and primers for LCN2 (sense; tgtatgccaccatctatgagc, antisense; tcctttagttccgaagtcagc), SLC22A17 (sense; gccattcgccactgctac, antisense; ggagaagagcccaaggacaga) and β-actin (ACTB) (sense; gacaggatgcagaaggagattact, antisense; tgatccacatctgctggaaggt) were used for RT-PCR as described previously [7]. In brief, 1 μg of total RNA was treated with 1 U/10 μl DNase I (Life Technologies). RT was performed using an RNA PCR kit (Takara Bio Inc., Otsu, Japan). The corresponding cDNA fragments were denatured at 94°C for 3 minutes, then subjected to 28 cycles of denaturing at 94°C for 10 seconds, annealing at 58°C for 10 seconds, and extension at 72°C for 20 seconds for LCN2, 30 cycles for SLC22A17 and 24 cycles for ACTB.

### Scratch Wound Healing Assay

The effects of LCN2 on cell migration were examined using the scratch wound healing assay with LCN2-silenced and control HHUA cells. In brief, 2 x 10^5^ cells were seeded onto 60-mm cell culture dishes. After cells reached approximately 70% confluence as a monolayer, the surface of the dishes were gently and slowly scratched linearly with a new 200-μl pipette tip, and the gap distance was then measured by photographs. After 24 hours, the migration distance was calculated by subtraction of the gap distance from the same point at 0 hour (immediately after scratching). Results were obtained from 3 independent experiments with 10 measurement points.

### WST-1 Assay

Cell viability and proliferation were evaluated using the WST-1 reagent (Roche Diagnostics, Basel, Switzerland) according to the manufacturer’s instructions. In brief, cells were seeded onto 96-well plates. After culturing the cells under various conditions, WST-1 reagent was added to the medium. After 2.5 hours, A450 wavelength light was measured using the microplate reader, Multiskan JX (Thermo Bioanalysis, Tokyo, Japan). Each result was obtained from 3 independent experiments with 16 replicates.

### Western Blotting

Proteins in the culture supernatant were extracted using the acetone precipitation method, as described previously [27]. Proteins extracted from 1 ml of culture supernatant and proteins extracted from cultured cells were subjected to a Western blot analysis, as described previously [28], using antibodies against human LCN2 (rat-monoclonal, clone # 220310, R & D systems, Minneapolis, MN), phospho-Akt (pAkt) (Ser473) (rabbit monoclonal, D9E, Cell Signaling Technology, Danvers, MA), Akt (rabbit monoclonal, C67E7, Cell Signaling Technology), phospho-MAPK (pMAPK) (Thr202/Tyr204) (rabbit monoclonal, D13.14.4E, Cell Signaling Technology), MAPK (rabbit monoclonal, 137F5, Cell Signaling Technology), p53 (mouse monoclonal, 1C12, Cell Signaling Technology), p21 (mouse monoclonal, EA10, Abcam, Cambridge, UK) and β-actin (mouse monoclonal, AC-15, BioMakor, Rehovot, Israel) as primary antibodies. The membranes were blotted with the primary antibody at 4°C overnight and then incubated with a peroxidase-conjugated secondary antibody. Bound antibodies were visualized using the ECL Western blot detection reagent (Amersham, Piscataway, NJ).

### H_2_O_2_ Treatment

Hydrogen peroxide (Wako Pure Chemical, Osaka, Japan) was added to the culture medium at various concentration. Cell viability was measured after 24 hours, by the WST-1 assay, as described above.

### Ultraviolet (UV) Irradiation

UV irradiation was performed as described previously [29]. Cells were seeded onto 96-well microplates (2 x 10^4^ cells/well). When cells attached to the bottom of plate, the culture medium was exchanged to serum-starved medium. After 12 hours, that microplate without a lid was irradiated by UV-C (wavelength of approximately 254 nm) using a UV germicidal lamp. Cell viability was then measured by the WST-1 assay. Our preliminary study showed that the most appropriate duration of UV-C irradiation was 15 minutes, and that the optimal time for performing the WST-1 assay was 10 hours after UV-C irradiation. In Western blotting, cells were cultured on 60-mm cell culture dishes, and harvested 8 hours after the UV treatment. The conditioned medium containing abundant LCN2 was collected as the supernatant of 24-hour cultured medium from control HHUA.

### Cisplatin (CDDP) Treatment

The effects of LCN2 on CDDP sensitivity were evaluated using the WST-1 assay. Cells were seeded onto 96-well microplates (2 x 10^3^ cells/well), and cultured in medium containing CDDP at various concentrations. Cell viability was measured after 48 hours for RL95-2 cells and 72 hours for HHUA and HEC1B cells by the WST-1 assay, as described above. In Western blotting, cells were cultured on 60-mm cell culture dishes, and harvested 36 hours after the CDDP treatment.

### Apostrand Assay

The effects of LCN2 on apoptosis were examined using the ApoStrand Assay (ApoStrand™ ELISA apoptosis detection kit; Enzo Life Sciences, NewYork, NY) according to the manufacturer's instructions. LCN2-silenced or control HHUA were seeded onto 96-well microplates and then treated with CDDP as described above. The ApoStrand assay was performed 48 hours after the CDDP treatment. Results were obtained from 3 independent experiments with 8 replicates.

## Statistical Analysis

Statistical analyses were conducted with Student’s t-test using the SPSS Statistics system (SPSS Inc., Chicago, IL).

## Results

### The Expression of LCN2 in Endometrial Carcinoma Cell Lines

**Fig 1:**
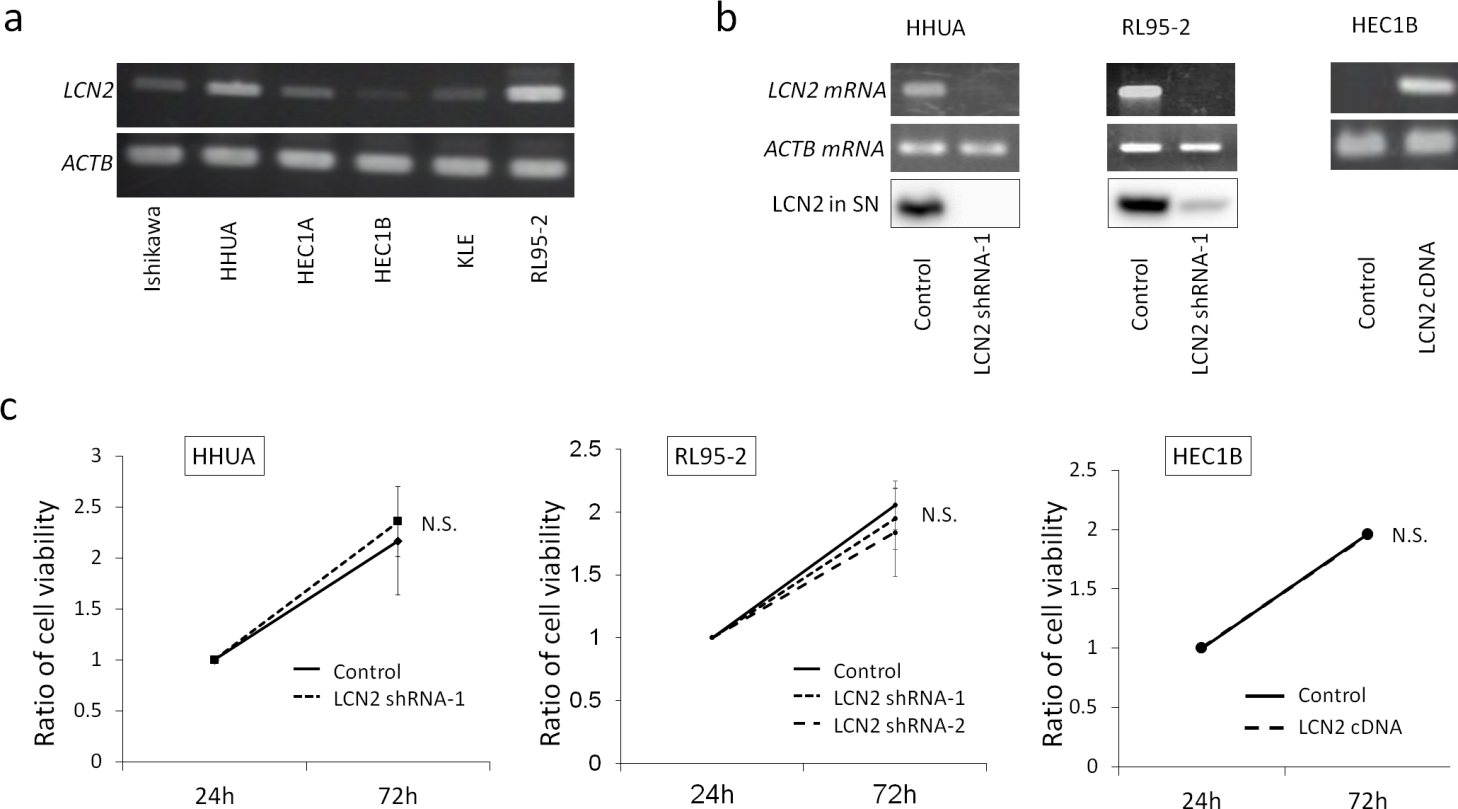
**a; Expressions of LCN2 and ACTB (internal control) in endometrial carcinoma cell lines by Semi-quantitative RT-PCR.** HHUA and RL95-2 strongly expressed LCN2, whereas Ishikawa and HEC1A moderately expressed it. **b; Expressions of LCN2 mRNA of endometrial carcinoma cell lines by RT-PCR, and the secreted LCN2 protein in the culture supernatant (SN) by Western blotting.** The expression of LCN2 in LCN2-silenced HHUA and RL95-2 (LCN2 shRNA-1) was markedly weaker than that in control cells (Control). The expression of LCN2 in LCN2-overexpressing HEC1B cells (LCN2 cDNA) was markedly stronger than that in control cells (Control). **c; Proliferation activity by WST-1 assay of HHUA, RL95-2 and HEC1B cells under normal culture conditions until 72 hours.** The results obtained indicated no significant difference in proliferation between LCN2-silenced cells (LCN2 shRNA-1 and 2) and control cells (Control) of HHUA and RL95-2, or between LCN2 overexpressing cells (LCN2 cDNA) and control cells (Control) of HEC1B.

The expression of LCN2 mRNA was the highest in HHUA and RL95-2 cells and lower in HEC1B cells among the endometrial carcinoma cell lines tested (Fig 1A). We established 4 clones of LCN2-silenced HHUA (LCN2 shRNA-1 to 4) using 4 types of shRNA. Also 2 clones of LCN2-silenced RL95-2 (LCN2 shRNA1 and 2) were established by 2 types of shRNA. The mRNA expression of LCN2 in LCN2-silenced HHUA and RL95-2 cells, and in LCN2 overexpressing HEC1B cells was confirmed by semiquantitative RT-PCR (Fig 1B, S2A Fig) and real-time PCR (data not shown). Western blotting combined with the acetone precipitation method revealed that the LCN2 protein was secreted into the culture supernatant of HHUA, RL95-2, and Ishikawa cells, with the highest level being observed in RL95-2 cells (S1 Fig). The expression of the LCN2 protein in the culture supernatant was markedly lower in LCN2-silenced HHUA and RL95-2 cells than in their control cells (Fig 1B).

### Effects of LCN2 on Proliferation

The WST-1 assay revealed no significant differences in the proliferation of LCN2-silenced cells and control cells of HHUA and RL95-2, and in LCN2-overexpressing cells and control cells of HEC1B until 72 hours of cultivation in medium containing 15% FBS (Fig 1C). These findings suggest that LCN2 do not affect the cell viability against cytotoxic stresses through the growth promotion effect in the following experiments.

### Effects of LCN2 on Cell Migration

**Fig 2:**
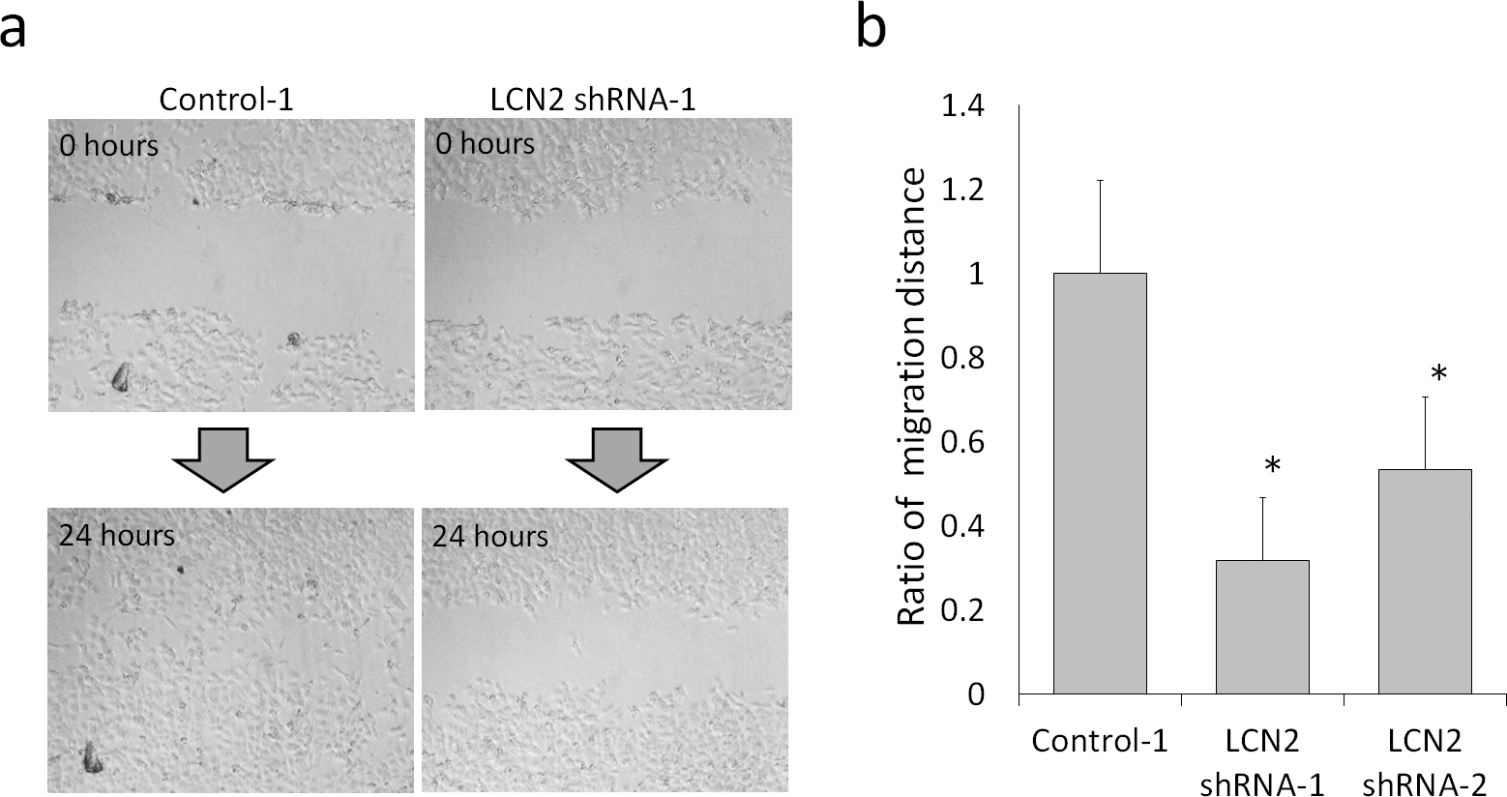
**a; Scratch wound healing assay.** Photomicrographs taken at 24 hours clearly showed that the scratch wound of control HHUA was narrower than that of LCN2-silenced HHUA. **b; Ratio of the migration distance in the scratch wound healing assay.** Two clones of LCN2-silenced HHUA cells were used for this assay. The migration distances of control HHUA cells (Control) were significantly longer than those of both LCN2-silenced HHUA cells (LCN2 shRNA-1 and 2). *; P<0.05

In the scratch wound healing assay, the migration distances of two LCN2-silenced HHUA clones (LCN2 shRNA-1 and 2) were significantly shorter than that of control HHUA cells (P < 0.05, by Student’s t-test) (Fig 2A and 2B), suggesting that LCN2 was involved in the migration of HHUA cells. After this experiment, the clone of HHUA LCN2 shRNA-1 was used as LCN2-silenced HHUA, because the expression of LCN2 in this clone was the lowest in the acquired clones.

### Effects of LCN2 on Cell Survival against Serum Starvation

**Fig 3:**
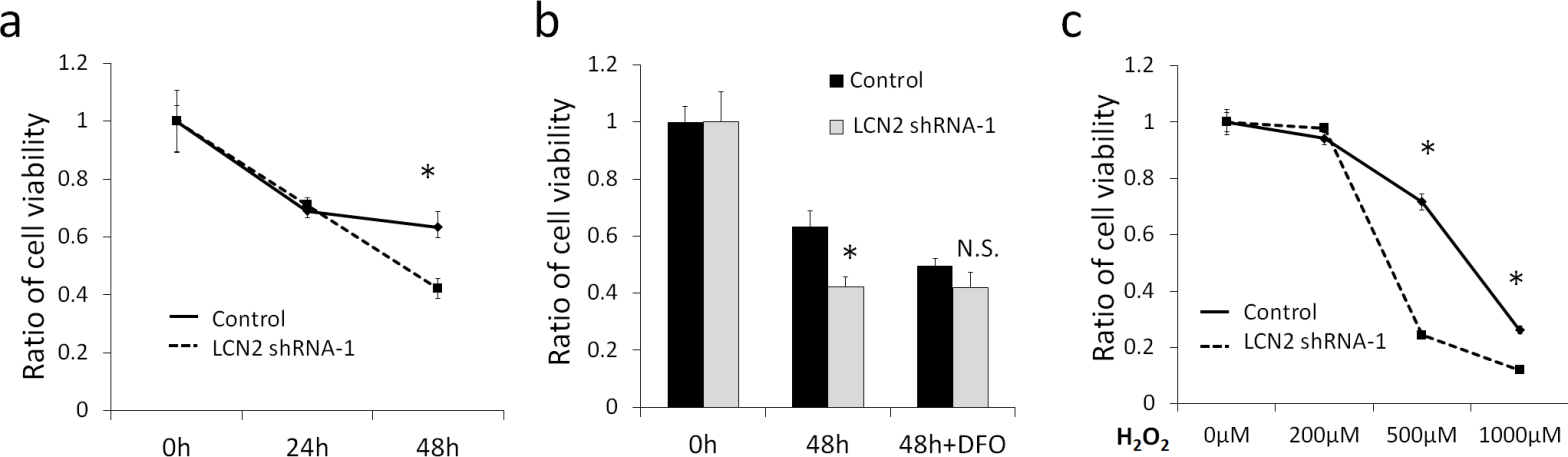
**a; WST-1 assay to measure survival activity under serum starvation conditions until 48 hours.** Decreases in the viability of LCN2-silenced HHUA were significantly greater than those in control HHUA at 48 hours. *; P<0.05. **b; The WST-1 assay under serum starvation conditions with or without 100μM deferoxamine (DFO) until 48 hours.** Differences in viability between both cells under serum starvation conditions were canceled by the addition of DFO. *; P<0.05, N.S. not significant. **c; WST-1 assay to measure survival activity under the H**_**2**_**O**_**2**_ **treatment at various concentrations until 24 hours.** Decreases in the viability of LCN2-silenced HHUA were significantly greater than those in control HHUA at a H_2_O_2_ concentration of 500 or 1000μM. *; P<0.05.

The effects of LCN2 on cell survival were then examined. When cells were cultured under 0.1% fetal bovine serum in the medium, the viabilities of control HHUA cells after 24 hours and 48 hours were approximately 69% and 63%, respectively (Fig 3A). The viability of LCN2-silenced HHUA after 24 hours was not significantly different from that of control HHUA. However, after 48 hours, the viability of LCN2-silenced HHUA was 42%, which was significantly lower than that of control HHUA. Therefore, we examined the effects of the iron chelator, deferoxamine (DFO), on cell survival. The addition of 100μM DFO reduced the cell viability of control HHUA at 48 hours, and canceled the difference in cell viability induced by LCN2 (Fig 3B). This result suggested that LCN2-mediated iron transport may support the survival of cells under serum starvation.

### Effects of LCN2 on Cell Survival against the H_2_O_2_ Treatment

The H_2_O_2_ treatment, which was used as oxidative stress, significantly reduced the cell viability of LCN2-silenced HHUA cells more than that of control HHUA cells (Fig 3C). The treatment with 500μM H_2_O_2_ reduced cell viability to 72% in LCN2-silenced HHUA and to 24% in control HHUA. This finding suggests that LCN2 may enhance the resistance against the oxidative stresses.

### Effects of LCN2 on Cell Survival against UV Irradiation

**Fig 4:**
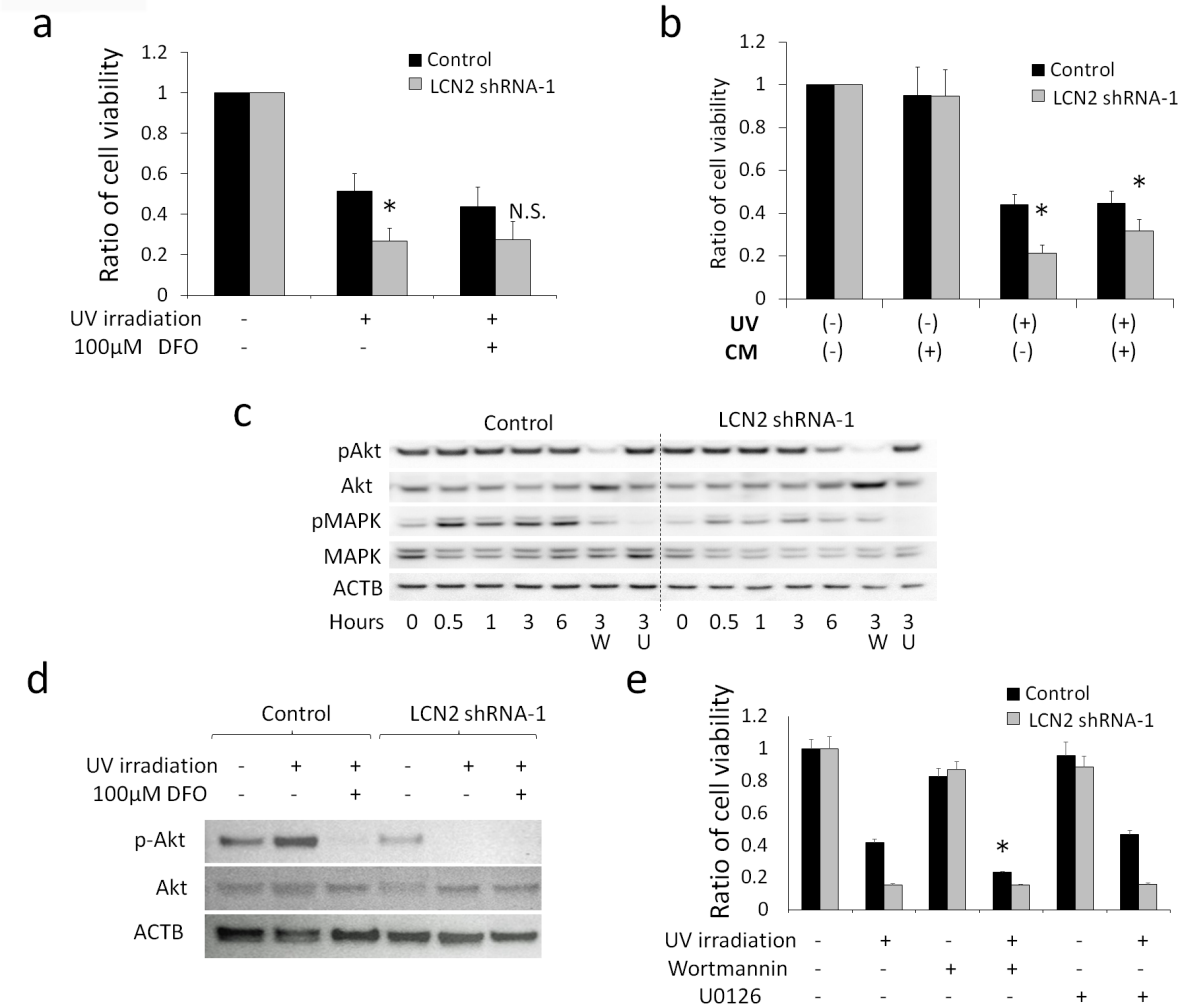
**a; WST-1 assay to measure survival activity 10 hours after ultraviolet (UV) irradiation with serum starvation.** Decreases in the viability of LCN2-silenced HHUA (LCN2 shRNA-1) were significantly greater than those in control HHUA (Control). The addition of deferoxamine (DFO) reduced this difference in cell viability. *; P<0.05, N.S.; not significant. **b; WST-1 assay after UV irradiation.** Each cell was cultured in conditioned medium (CM) collected from control HHUA (CM: +). The viability of LCN2-silenced HHUA after UV irradiation was increased by CM. The difference in viability after UV was diminished by CM. *; P<0.05 **c; Western blotting: Changes over time in the expression of phosphorylated Akt (pAkt), Akt, phosphorylated MAPK (pMAPK), and MAPK after UV irradiation.** The expression of pAkt and pMAPK in LCN2-silenced HHUA 6 hours after UV was apparently diminished. pMAPK expression appeared to have cooperated with pAkt expression. The PI3K inhibitor also reduced the expression of pMAPK. Number: Time (hours) after UV irradiation, W: add 1 μM wortmannin (PI3K inhibitor) to the medium, U: add 10 μM U0126 (MEK inhibitor) to the medium. ACTB; β-actin as an internal control. **d; Western blotting, 8 hours after UV irradiation.** The expression of pAkt in control HHUA was appeared to be diminished by the addition of DFO. **e; WST-1 assay after UV irradiation.** Cells were cultured in serum-starved medium containing 1 μM wortmannin or 10 μM U0126. The addition of wortmannin reduced the survival of control HHUA. *; significantly different from UV irradiation without wortmannin and U0126 in control HHUA (P<0.05).

Cell viability was reduced to approximately 51% in control HHUA (Fig 4A) 10 hours after UV irradiation. The viability of LCN2-silenced HHUA (LCN2 shRNA-1) after UV irradiation was 27%, which was significantly lower than that of control HHUA. This difference in cell viability was decreased by the addition of 100μM DFO (Fig 4A). Similar results were obtained using other shRNA clones of LCN2-silenced HHUA (LCN2 shRNA-2, 3 and 4) (S2B Fig). These results suggested that LCN2 was involved in the iron-dependent survival of HHUA after UV-C irradiation. Since LCN2 is a secretory protein, the effects of conditioned medium obtained from control HHUA on the survival of LCN2-silenced HHUA were examined. The conditioned medium collected from control HHUA increased the viability of LCN2-silenced HHUA (LCN2 shRNA-1 and 2) after UV irradiation, and decreased differences in viability between both cells (Fig 4B, S2C Fig).

In order to elucidate the mechanisms underlying LCN2-induced UV irradiation resistance, the involvement of PI3K and MAPK pathways was examined by Western blotting. The expression of pAkt and pMAPK was detected in both cells up to 3 hours after UV irradiation. However, these expression levels decreased in LCN2-silenced HHUA 6 hours after UV irradiation (Fig 4C). The expression of pMAPK was similar to that of pAkt and was decreased by the PI3K inhibitor. Although the expression of pAkt in control HHUA 8 hours after UV irradiation was stronger than that before UV irradiation, its expression in LCN2-silenced HHUA was diminished (Fig 4D). The addition of DFO decreased the expression of pAkt, even in control HHUA. The difference in cell viability between both cells was diminished by the addition of the PI3K inhibitor, wortmannin (1μM) (Fig 4E), but not by the MEK inhibitor, U0126 (20μM). These results suggest that LCN2 is involved in cell survival after UV-C irradiation via the PI3K pathway, and iron may be an important factor in this process.

### Effects of LCN2 on Cell Survival against the CDDP Treatment

**Fig 5:**
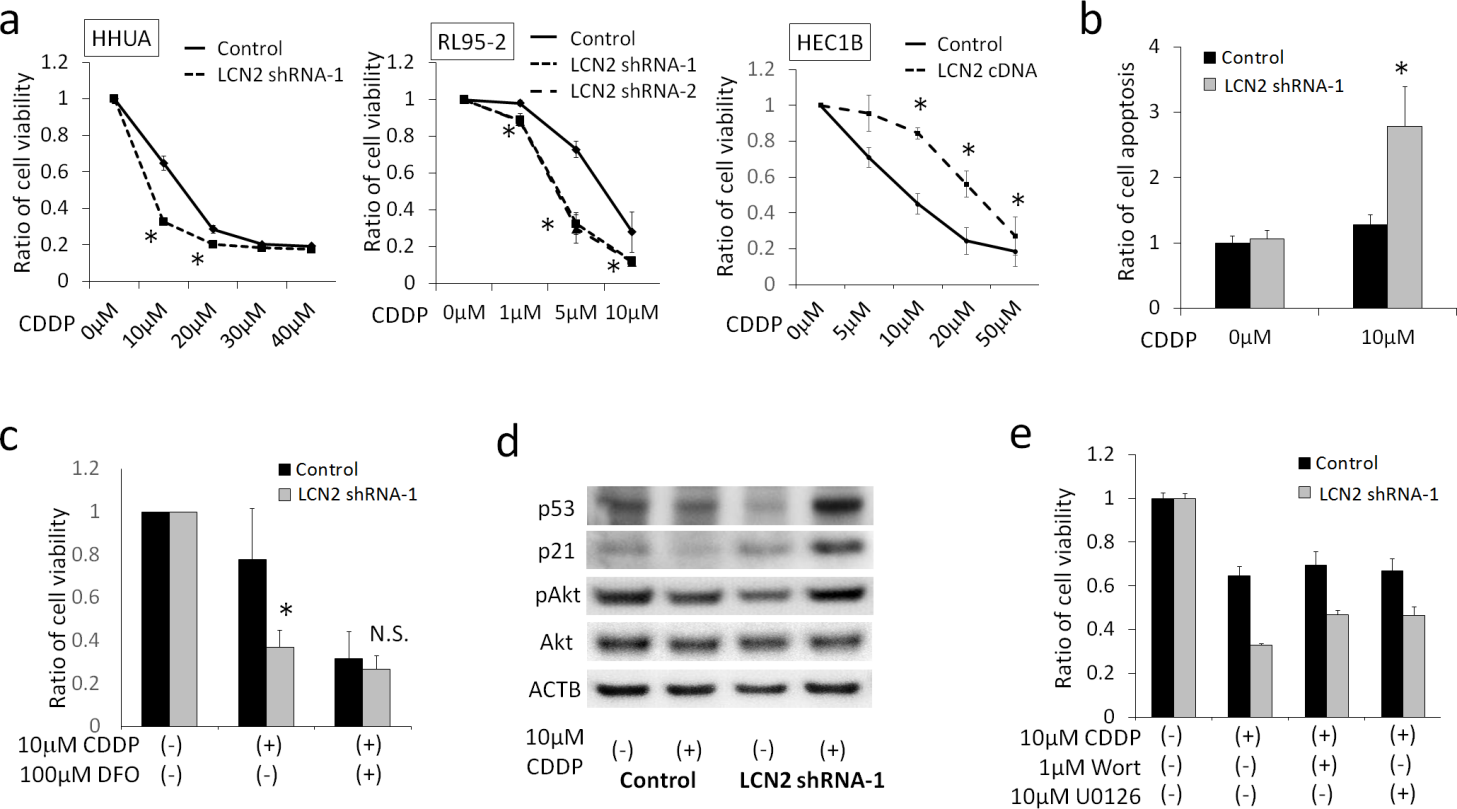
**a; WST-1 assay to measure cisplatin (CDDP) sensitivity in HHUA, RL95-2 and HEC1B cells.** Decreases in the viability of LCN2-silenced cells (LCN2 shRNA-1 and 2) were significantly greater than those in the control cells (Control) in both HHUA and RL95-2. Decreases in the viability of LCN2-overexpressing cells (LCN2 cDNA) were significantly smaller than those in the control cells (Control) in HEC1B. *; significantly lower than the control (P<0.05). **b; Apostrand assay.** Apoptosis was significantly enhanced in LCN2-silenced HHUA. *; P<0.05. **c; WST-1 assay.** The difference in viability between both cells under the CDDP treatment was canceled by the addition of DFO. *; P<0.05, N.S.; not significant. **d; Western blotting under the CDDP treatment.** No apparent change in pAkt expression was observed. The strong expression of p53 and p21 was observed in LCN2-silenced HHUA under the CDDP treatment. **e; WST-1 assay under the CDDP treatment.** Neither wortmannin nor U0126 affected the viability of control HHUA, and slightly increased the viability of LCN2-silenced HHUA.

The effects of LCN2 on CDDP sensitivity were then examined. Decreases in the viability of LCN2-silenced HHUA (LCN2 shRNA-1) with the 10μM CDDP treatment (33%) were significantly greater than those in control HHUA (65%, P<0.05) (Fig 5A). Increase of CDDP sensitivity was also observed in other shRNA clones of HHUA (LCN2 shRNA-2, 3 and 4) (S2D Fig). A similar effect of LCN2 was observed in RL95-2 cells. Decreases in the viability of LCN2-silenced RL95-2 with the 5μM CDDP treatment (29% to 33%) were significantly greater than those in control RL95-2 (73%, P<0.05) (Fig 5A). In contrast, decreases in the viability of LCN2-overexpressing HEC1B with the 10μM CDDP treatment (84%) were significantly smaller than those in control HEC1B (45%, P<0.05) (Fig 5A). Apoptosis induced by the 10μM CDDP treatment was detected more frequently in LCN2-silenced HHUA than in control HHUA (P<0.05) (Fig 5B). This difference in cell viability under the 10μM CDDP treatment was canceled by the addition of 100μM DFO (Fig 5C).

**Fig 6:**
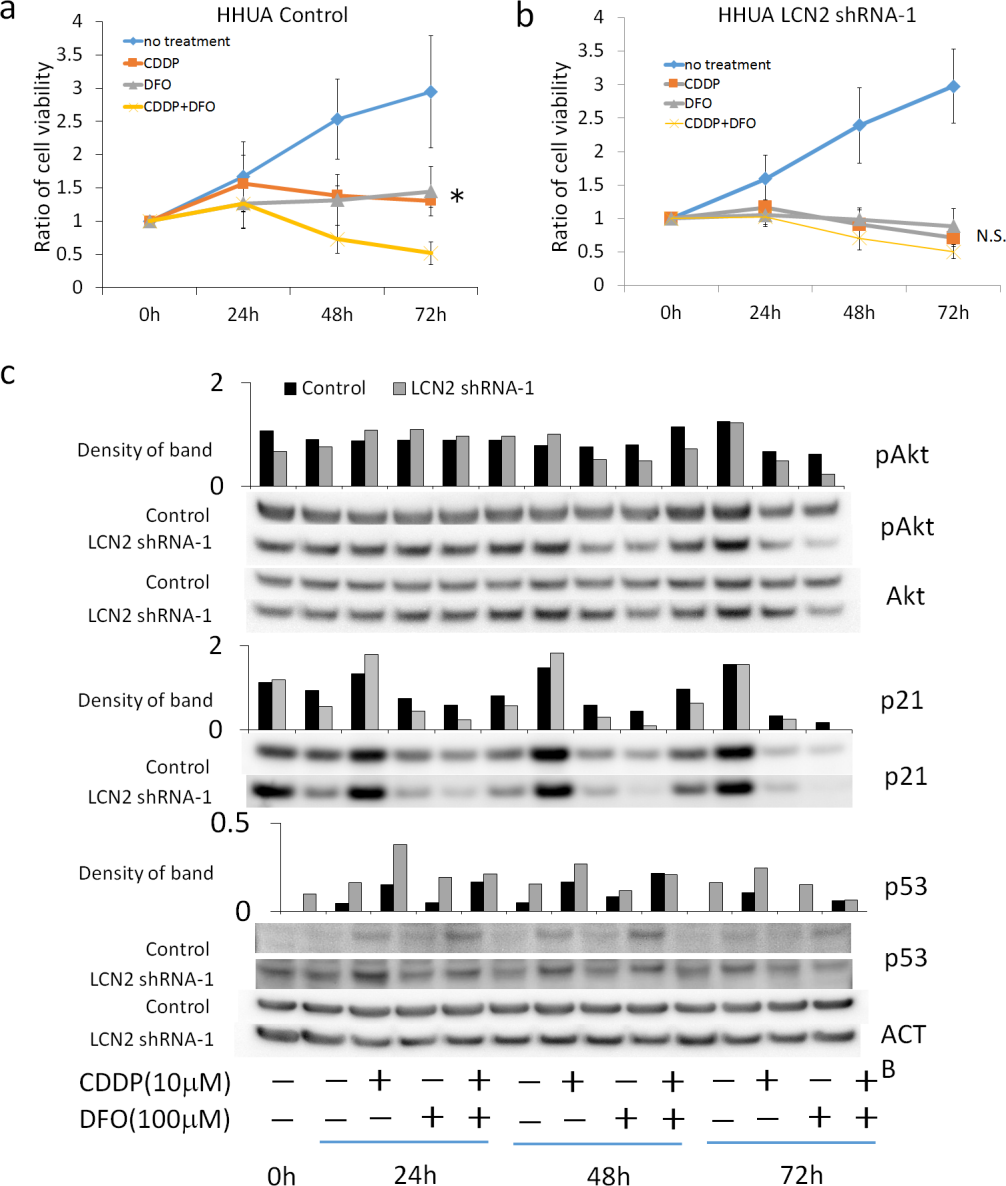
**a; Cell viability of HHUA Control cells treated with CDDP and/or DFO. (WST-1 assay)** *: The viability of cells treated with CDDP and DFO was significantly lower than that treated with each drug at 72 hours. (P<0.05) **b; Cell viability of HHUA LCN2 shRNA-1 cells treated with CDDP and/or DFO. (WST-1 assay)** N.S.: The viability of cells was not different between the treatment with both drugs and that with single drug. **c; The expression of pAkt, Akt, p21 and p53 in HHUA Control and LCN2 shRNA-1 cells at 24, 48 and 72 hours of CDDP/DFO treatment (Western blotting).** The density of each band was measured by densitometry. ACTB (beta-actin) was used as internal control. Graphs indicated the ratio of density compared with ACTB.

The involvement of the PI3K-Akt pathway was examined to investigate the intracellular mechanisms underlying LCN2-mediated cisplatin resistance. The results of Western blotting showed that the expression of pAkt was not affected by LCN2-silencing under the CDDP treatment (Fig 5D), whereas the expression of p53 and p21 in LCN2-silenced HHUA was higher than that in control HHUA. Furthermore, the viability of control HHUA under the 10μM CDDP treatment was not decreased by the addition of wortmannin or U0126 (Fig 5E). To clarify the effect of DFO on LCN2-induced CDDP resistance, cell viability and the expressions of pAkt, p21 and p53 were examined at 24h, 48h and 72h after the treatment with CDDP and DFO (Fig 6). DFO inhibits cell proliferation in a single agent, and almost completely canceled the difference of CDDP-resistance between Control HHUA and LCN2-silenced HHUA cells (Fig 6A and 6B). No difference of the expressions of pAkt was observed between Control and LCN2-silenced HHUA cells with CDDP treatment. DFO treatment also suppressed the expression of pAkt induced by CDDP (Fig 6C) as well as by UV (Fig 4C). The suppression of pAkt by DFO was stronger in LCN2-silenced cells than in Control cells. The expression of p21 was enhanced by CDDP treatment, particularly in LCN2-silenced cells at 24 and 48 hours. DFO also markedly suppressed the expression of p21 induced by CDDP. These results suggested that LCN2 enhanced the survival of HHUA cells under the CDDP treatment via the iron-mediated downregulation of p53 and p21, but not the PI3K pathway or MAPK pathway. LCN2 might mediate the expression of p53-p21 axis and pAkt via iron-uptake, and DFO down-regulated these proteins. Iron-depletion by DFO might induce apoptosis through the suppression of PI3K pathway.

## Discussion

We herein demonstrated that LCN2-silencing in HHUA significantly diminished the migration activity of HHUA cells. LCN2-mediated cell migration has been reported in other tumors such as breast and prostate carcinomas [30, 31]. The results obtained in the present study were consistent with these findings. The underlying mechanism of LCN2-induced migration has not yet been elucidated in detail; however, Hu et al. reported that LCN2-induced migration was triggered by detachment from adjacent cells via the down-regulation of E-cadherin [32]. LCN2-mediated migration has also been associated with alterations in the cellular structure such as lamellipodia at the leading edge via Rac1. Lin HH et al. showed that the migration of the endometrial carcinoma cell RL95-2 was stimulated by LCN2, and was mediated by IL-8, indicating that LCN2 helped cancer cells to escape local inflammation to survive [33]. Ding et al recently reported that LCN2-induced cell migration was caused by the up-regulation of SLUG, and this was mediated by the Erk1/2 pathway in prostate carcinoma [31]. Collectively, LCN2-induced migration, together with increases in collagenase and MMP activity, accelerates invasion potential, which is supported by our previous finding that the strong expression of LCN2 with its receptor was observed in endometrial carcinoma, especially, at the invasion front in the myometrium [17]. These findings suggest that LCN2 markedly accelerates the invasion potential of endometrial carcinoma cells.

The acquisition of resistance against various stressors is important for the progression and persistence of carcinoma cells. The present study revealed that LCN2 positively regulated the survival of HHUA cells under stresses including serum starvation, H_2_O_2_ treatment, UV irradiation, and CDDP treatment. The viability of LCN2-silenced HHUA cells decreased after a 48-hour culture under serum-starved conditions, suggesting that LCN2 suppressed serum starvation-induced apoptosis. In thyroid cancer cells, serum withdrawal evoked apoptosis, and this was suppressed by the addition of conditioned medium containing LCN2 [34]. Our results are consistent with these findings. In contrast, Lin et al showed that the addition of LCN2 to the culture medium for endometrial carcinoma RL95-2 cells under serum-starved conditions decreased cell viability [33]. Another study reported that the knockdown of LCN2 increased the survival of hepatocellular carcinoma cells [35]. These findings suggested the bi-functional aspect of LCN2; however, the reason for this discrepancy currently remains unknown.

In the present study, the viability of LCN2-silenced HHUA cells decreased after a 24-hour culture under the H_2_O_2_ treatment. This result suggested that LCN2 contributed to cell survival against the H_2_O_2_ treatment as an oxidative stress, because H_2_O_2_ is deeply involved in producing hydroxyl radical which is one of the major reactive oxygen species.

Furthermore, our results revealed that tolerance against UV irradiation was enhanced by LCN2. UV irradiation generates reactive oxygen species (ROS) in cells, which induce apoptosis in tumors, thereby killing malignant cells [36]. A recent study revealed that the expression of LCN2 was induced by various stresses including ROS [37] and LCN2 suppressed the toxic effects of ROS and subsequent DNA damage in various cells, thereby protecting cells [38–40]. This is the first study to show LCN2-induced UV irradiation resistance, and we speculated that LCN2 facilitated the scavenging of ROS-induced DNA damage and, thus, suppressed apoptosis, resulting in prolonged cell survival. γ-irradiation also generates ROS and kills tumor cells, similar to UV irradiation. Therefore, we speculated that LCN2 promoted radiation resistance. The present study also indicated that LCN2-mediated UV irradiation resistance correlated with activation of the PI3K-Akt pathway, which is a potent anti-apoptotic and survival signal cascade [41]. Chung et al. recently reported that the enhanced expression of LCN2 led to the phosphorylation of PI3K-Akt and JNK in human aortic smooth muscle cells [42]. In contrast, the function of LCN2 was previously shown to be exerted partly through blockage of the JNK and PI3K pathways in hepatocellular carcinoma cells [35]. Cancer stem cells are known to be associated with low ROS levels [36]. Therefore, the elevated expression of LCN2 may confer a stem cell nature to tumor cells, possibly via the PI3K-Akt pathway. Further studies are needed to clarify the relationship between the expression of LCN2 and stemness of endometrial carcinoma cells.

The present study also indicated that LCN2 increased the resistance of endometrial carcinoma cells, HHUA, RL95-2 and HEC1B, to CDDP. In addition, we observed that Ishikawa cells overexpressing SLC22A17, a LCN2 receptor, increased resistance to CDDP (data not shown). LCN2 has been shown to promote gemcitabine resistance in pancreatic adenocarcinoma cells *in vitro* and *in vivo* [43]. In contrast, resistance to 1, 3-Bis (2-chloroethyl)-1-nitrosourea (BCNU), an alkylating agent commonly used for glioblastoma, was associated with the reduced expression of LCN2 in glioblastoma cells [44]. To the best of our knowledge, this is the first study to show LCN2-induced cisplatin resistance in endometrial carcinoma. Although the mechanism underlying LCN2-mediated chemo resistance has not yet been elucidated, Leung et al indicated that pancreatic cancer cells that weakly expressed LCN2 had higher levels of cleaved caspase 3[43]. Furthermore, LCN2-induced gemcitabine resistance has been associated with increases in the expression of anti-apoptotic genes such as BIRC2 and decreases in the expression of pro-apoptotic genes such as AIFM1 [43]. In the present study, we initially expected the PI3K pathway to be involved in the survival of HHUA cells against the CDDP treatment, as observed in UV irradiation. However, no significant difference was observed in the expression of pAkt between control and LCN2-silenced HHUA cells, and the PI3K inhibitor did not reduce the viability of control HHUA cells. On the other hand, the PI3K and MEK inhibitors slightly increased the viability of LCN2-silenced HHUA cells under the CDDP treatment. Therefore, the PI3K and MAPK pathways may be partly involved in cell death induced by the CDDP treatment in LCN2-silenced cells. This study also demonstrated that LCN2-induced cisplatin resistance was associated with the down-regulated expression of p53-p21. P53-p21 is a pivotal axis that regulates the cell cycle and apoptosis in mammalian cells [45, 46]. The relationship between the overexpression of LCN2 and reductions in the expression of p53-p21 was previously reported in cervical carcinoma cells [47]. Thus, the cisplatin-induced, LCN2-mediated loss of the intrinsic brake system results in prolonged cell survival.

This study demonstrated that the above described LCN2 functions such as the promotion of invasive activity and resistance against serum starvation, UV irradiation, and cisplatin, were iron-dependent. The 24p3 and 24p3 receptors have been shown to regulate the influx and/or efflux of iron in order to control intracellular iron concentrations [11]. In thyroid carcinoma cells, the increases in apoptosis through the knockdown of LCN2 were found to be canceled by the addition of Ferric ions or iron-loaded transferrin [34]. These findings suggest that adequate intracellular iron concentrations may be important for the onset of these functions. This aspect may be of particular importance in endometrial carcinoma because normal endometrial glands, from which endometrial carcinoma arise, are exposed to blood during menstrual shedding. The occurrence of endometrial carcinoma in patients with hematometra was also reported [48].

## Conclusions

Our results revealed that LCN2 was involved in promoting migration and survival against UV irradiation and cisplatin. In addition, LCN2-induced prolonged cell survival was mediated by multiple pathways including PI3K/Akt and p53-p21. These LCN2 functions conferred malignant potential to endometrial carcinoma cells. Therefore, LCN2 may be a potential molecular target for the treatment of endometrial carcinoma.

## Supporting Information

S1 Fig Western blotting of the LCN2 protein in the culture supernatant. The expression of the LCN2 protein was detected in HHUA, RL95-2, and Ishikawa, and was particularly abundant in RL95-2.
(PPTX)

S2 Fig **a; Cell viability of HHUA Control cells treated with CDDP and/or DFO. (WST-1 assay) b; Cell viability of HHUA LCN2 shRNA-1 cells treated with CDDP and/or DFO. (WST-1 assay) c; The expression of pAkt, Akt, p21 and p53 in Control and LCN2-silenced HHUA cells at 24, 48 and 72 hours of CDDP/DFO treatment (Western blotting).** ACTB (beta-actin) was used as internal control. The expression of p21 at 24 and 48 hours in LCN2 shRNA-1 with CDDP treatment was as the same as that in Control with CDDP; however, the expression of ACTB in LCN2 shRNA-1 was weaker than that in Control.
(PDF)
