# Supplementary material for: Lipocalin 2 Enhances Migration and Resistance against Cisplatin in Endometrial Carcinoma Cells
Source: PLoS One. 2016 May 11;11(5):e0155220. doi: 10.1371/journal.pone.0155220 (PMC4864227; doi:10.1371/journal.pone.0155220)
Supplement: S1 Fig — The expression of the LCN2 protein was detected in HHUA, RL95-2, and Ishikawa, and was particularly abundant in RL95-2. (PPTX) [file pone.0155220.s001.pptx]

## Slide 1
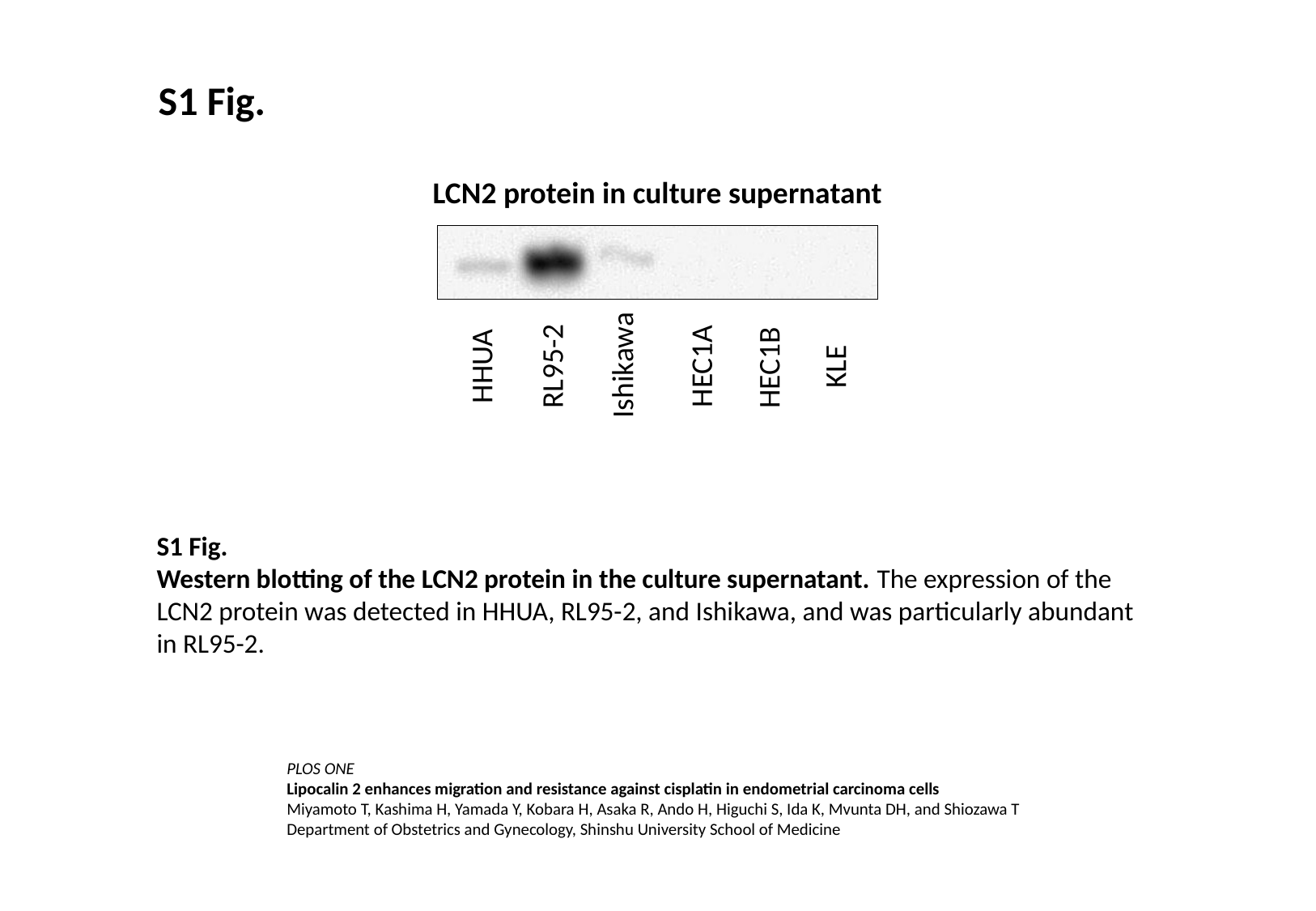

S1 Fig.
LCN2 protein in culture supernatant
Ishikawa
HEC1A
HHUA
RL95-2
KLE
HEC1B
S1 Fig.
Western blotting of the LCN2 protein in the culture supernatant. The expression of the LCN2 protein was detected in HHUA, RL95-2, and Ishikawa, and was particularly abundant in RL95-2.
PLOS ONE
Lipocalin 2 enhances migration and resistance against cisplatin in endometrial carcinoma cells
Miyamoto T, Kashima H, Yamada Y, Kobara H, Asaka R, Ando H, Higuchi S, Ida K, Mvunta DH, and Shiozawa T
Department of Obstetrics and Gynecology, Shinshu University School of Medicine
