# Supplementary material for: Lipocalin 2 Enhances Migration and Resistance against Cisplatin in Endometrial Carcinoma Cells
Source: PLoS One. 2016 May 11;11(5):e0155220. doi: 10.1371/journal.pone.0155220 (PMC4864227; doi:10.1371/journal.pone.0155220)
Supplement: S2 Fig — a; Cell viability of HHUA Control cells treated with CDDP and/or DFO. (WST-1 assay) b; Cell viability of HHUA LCN2 shRNA-1 cells treated with CDDP and/or DFO. (WST-1 assay) c; The expression of pAkt, Akt, p21 and p53 in Control and LCN2-silenced HHUA cells at 24, 48 and 72 hours of CDDP/DFO treatment (Western blotting). ACTB (beta-actin) was used as internal control. The expression of p21 at 24 and 48 hours in LCN2 shRNA-1 with CDDP treatment was as the same as that in Control with CDDP; however, the expression of ACTB in LCN2 shRNA-1 was weaker than that in Control. (PDF) [file pone.0155220.s002.pdf]

S2 Fig.

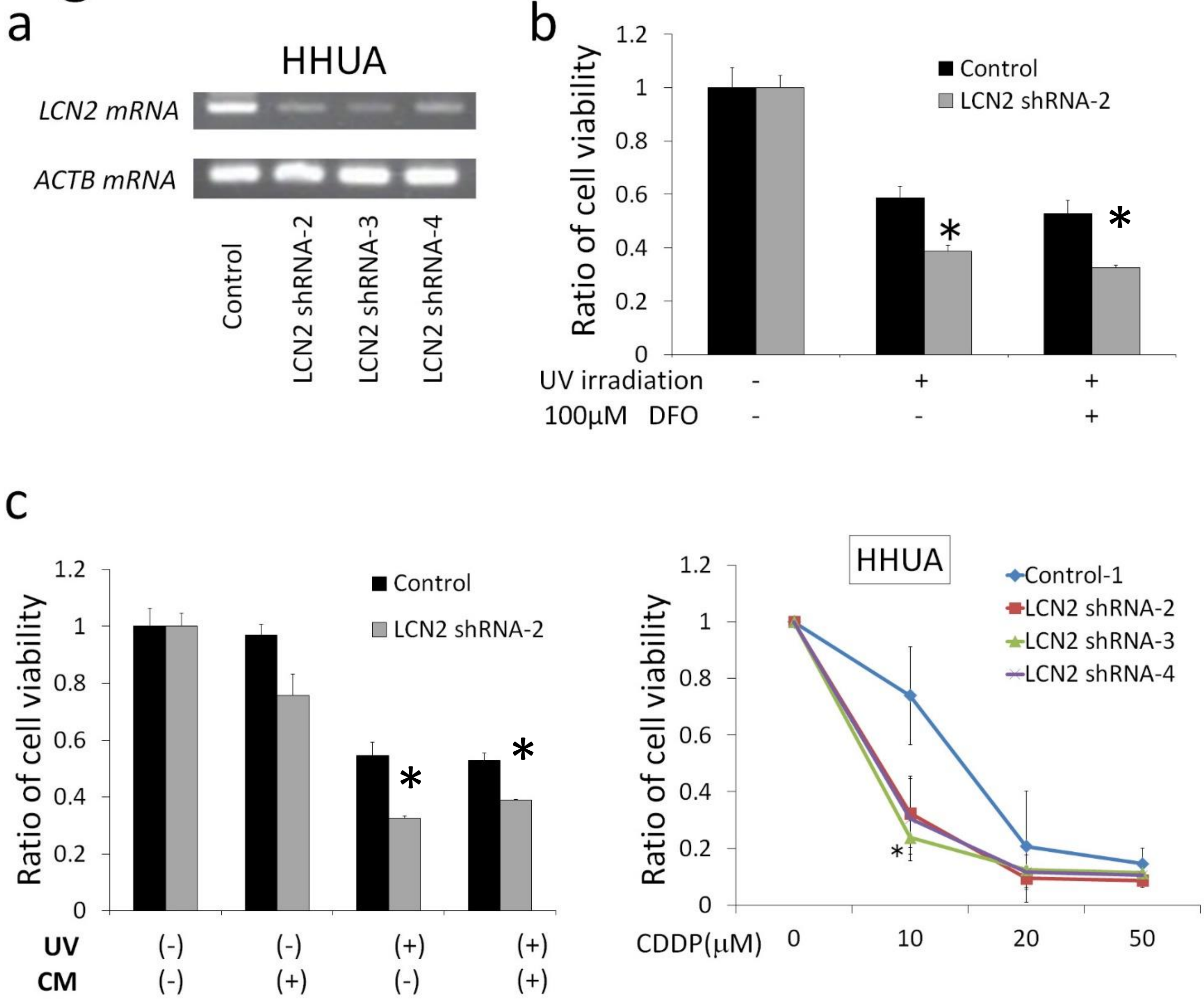

S2 Fig. a; Expressions of LCN2 and ACTB (internal control) in HHUA Control and its LCN2-silenced clones (LCN2 shRNA-2, 3 and 4). (Semi-quantitative RT-PCR) **b; WST-1 assay to measure survival activity 10 hours after ultraviolet (UV) irradiation with serum starvation.** Decreases in the viability of LCN2-silenced HHUA (LCN2 shRNA-2) were significantly greater than those in control HHUA (Control). The addition of deferoxamine (DFO) further reduced cell viability. *; P<0.05. **c; WST-1 assay after UV irradiation.** Each cell was cultured in conditioned medium (CM) collected from control HHUA (CM: +). The viability of LCN2-silenced HHUA (LCN2 shRNA-2) after UV irradiation was partially recovered by CM. *; P<0.05 **d; WST-1 assay to measure cisplatin (CDDP) sensitivity in HHUA.** Decreases in the viability of LCN2-silenced cells (LCN2 shRNA-2, 3 and 4) were significantly greater than those of HHUA Control cells (Control). *; P<0.05.
